# Lymph node metastasis in early invasive lung adenocarcinoma: Prediction model establishment and validation based on genomic profiling and clinicopathologic characteristics

**DOI:** 10.1002/cam4.70039

**Published:** 2024-07-24

**Authors:** Wei Guo, Tong Lu, Yang Song, Anqi Li, Xijia Feng, Dingpei Han, Yuqin Cao, Debin Sun, Xiaoli Gong, Chengqiang Li, Runsen Jin, Hailei Du, Kai Chen, Jie Xiang, Junbiao Hang, Gang Chen, Hecheng Li

**Affiliations:** ^1^ Department of Thoracic Surgery Ruijin Hospital, Shanghai Jiao Tong University School of Medicine Shanghai China; ^2^ Department of Thoracic Surgery Huashan Hospital, Fudan University Shanghai China; ^3^ Department of Pathology Ruijin Hospital, Shanghai Jiao Tong University School of Medicine Shanghai China; ^4^ Genecast Biotechnology Co., Ltd Wuxi China

**Keywords:** lung adenocarcinoma, lymph node metastasis, next‐generation sequencing, prediction model

## Abstract

**Background:**

The presence of lymph node (LN) metastasis directly affects the treatment strategy for lung adenocarcinoma (LUAD). Next‐generation sequencing (NGS) has been widely used in patients with advanced LUAD to identify targeted genes, while early detection of pathologic LN metastasis using NGS has not been assessed.

**Methods:**

Clinicopathologic features and molecular characteristics of 224 patients from Ruijin Hospital were analyzed to detect factors associated with LN metastases. Another 140 patients from Huashan Hospital were set as a test cohort.

**Results:**

Twenty‐four out of 224 patients were found to have lymph node metastases (10.7%). Pathologic LN‐positive tumors showed higher mutant allele tumor heterogeneity (*p* < 0.05), higher tumor mutation burden (*p* < 0.001), as well as more frequent KEAP1 (*p* = 0.001), STK11 (*p* = 0.004), KRAS (*p* = 0.007), CTNNB1 (*p* = 0.017), TP53, and ARID2 mutations (both *p* = 0.02); whereas low frequency of EGFR mutation (*p* = 0.005). A predictive nomogram involving male sex, solid tumor morphology, higher T stage, EGFR wild‐type, and TP53, STK11, CDKN2A, KEAP1, ARID2, KRAS, SDHA, SPEN, CTNNB1, DICER1 mutations showed outstanding efficiency in both the training cohort (AUC = 0.819) and the test cohort (AUC = 0.780).

**Conclusion:**

This study suggests that the integration of genomic profiling and clinical features identifies early‐invasive LUAD patients at higher risk of LN metastasis. Improved identification of LN metastasis is beneficial for the optimization of the patient's therapy decisions.

## 1 INTRODUCTION

Lung cancer is the leading cause of cancer‐associated mortality worldwide, of which nonsmall cell lung cancer (NSCLC) is one of the two major types, accounting for 85% of lung cancers.^1^ Accurate lymph node (LN) staging is essential for NSCLC. Patients who have no LN metastasis typically receive local treatments. This often involves resection or, for those with limited cardiopulmonary function, radical radiotherapy. For patients with LN metastases, it has become apparent that downstaging of mediastinal LNs followed by complete surgical resection could improve long‐term survival.^2^, ^3^ Compared with other subtypes of NSCLC, lung adenocarcinoma (LUAD) is associated with a higher risk of occult LN metastasis,^4^, ^5^ ranging from 7.4% to 36.6%.^6^, ^7^ Of clinical importance, the 2‐year recurrence rate was higher in the false negative patients than in true negative patients (60% vs. 21%).^8^ Thus, developing an accurate and noninvasive preoperative methodology for assessing LN status is of paramount importance. This is essential for the selection of an appropriate clinical treatment strategy for LUAD patients and the accurate evaluation of their prognostic outcomes.

At present, positron emission tomography‐computed tomography (PET/CT) imaging is the standard noninvasive examination to detect LN metastases in clinical practice^9^, ^10^; however, this method still presents a high false positive rate.^11^ In patients without fluorodeoxyglucose uptake for PET/CT, the incidence of pathological mediastinal LN metastases may range from 13.2% to 24.1%.^8^, ^11^ Some clinical features have also been revealed to be associated with LN metastases, including centrally located tumors, large tumor size, high maximum standardized uptake value (SUVmax) of the primary tumor, and components of micropapillary and solid subtypes.^6^, ^7^, ^12^, ^13^ In order to better detect LN metastasis, several studies have attempted to develop predictive models of clinical features with or without PET‐CT; however, these models either lack validation, contain complex parameters that are difficult to apply clinically, or have unsatisfactory efficacy.^14^, ^15^, ^16^

In the field of clinical medicine, the widespread adoption of comprehensive next‐generation sequencing (NGS) is enhancing our understanding of tumor biology. This advanced technique is pivotal in pinpointing alterations in key driver mutations that can be targeted for treatment, as well as in assessing the prognosis of patients suffering from NSCLC.^17^ Recent studies utilizing NGS revealed that various genotypes of NSCLC exhibit distinct tendencies to progress to LN metastasis.^18^, ^19^ Since LN metastasis has a more vital impact on treatment strategies for early‐stage lung cancer, and the occurrence of LN metastasis in smaller tumors (3 cm or less) might offer a more realistic representation of genetic mutations, we investigated the genomic profiling of T1 LUAD, and constructed a prediction model using screeded out features with LN metastasis.

## 2 MATERIALS AND METHODS

### 2.1 Patients

The study included 364 patients diagnosed with pathological T1 (pT1) LUAD between 2018 and 2022; 224 of them were from Ruijin Hospital, and the other 140 were from Huashan Hospital. Only patients who received systematic mediastinal LN dissection (six stations) were enrolled. Blood and tumor tissue samples were collected from each patient. Clinical characteristics such as sex, age, tumor location, smoking status, computed tomography manifestations, and pathological features were meticulously reviewed and extracted from medical records. This study was approved by the Ethics Committees of Ruijin Hospital (July 2, 2021, NO.2021–219) and Huashan Hospital (March 3, 2021, NO.2021–115). Written informed consent was obtained from all patients. All processes strictly adhered to the guidelines of the Ethics Committee and were in accordance with the principles of the Declaration of Helsinki. Informed consent or a waiver was obtained from each patient.

### 2.2 Tumor genomic analysis

Formalin‐fixed, paraffin‐embedded blocks of lung cancer tissues were used to obtain neoplastic cellularity of ≥20% for genomic DNA extraction using the TIANamp Genomic DNA Kit (TIANGEN, China). Genomic DNA from peripheral blood lymphocytes (PBL) was extracted using the TGuide S32 Magnetic Blood Genomic DNA Kit (TIANGEN, China). DNA concentrations were measured using the Qubit dsDNA High Sensitivity Assay Kit (Thermo Fisher Scientific, Waltham, MA, USA), and DNA quality was assessed using the Agilent 2100 BioAnalyzer (Santa Clara, CA, USA). Genomic DNA (30–300 ng) was extracted from either tumor tissue or PBL sample and sheared with Covaris LE220 to a length of 200 bp using the recommended settings. According to the manufacturer's instructions, fragmented DNA was used to construct a library using the KAPA Hyper Preparation Kit (Kapa Biosystems, MA, USA). All libraries were quantified using the AccuGreen High Sensitivity dsDNA Quantitation Kit (Biotium, CA, USA), and the sizes of the libraries were determined using the Agilent Bioanalyzer 2100 (Agilent, CA, USA). Target DNA regions were captured using the HyperCap Target Enrichment Kit (Roche, Switzerland). DNA was then hybridized into a specially designed Genescope panel spanning 769 cancer‐related genes (Table S1). The final sequencing libraries were quantified using the Qubit dsDNA HS Assay Kit (Thermo Fisher Scientific, MA, USA). The samples were then processed using the Illumina NovaSeq 6000 system (Illumina, CA, USA) for paired‐end sequencing. After executing data quality control using Trimmomatic (v0.36),^20^ reference mapping was performed using the BWA aligner (v0.7.17),^21^ and duplication masking was performed using Picard (v2.23.0).^22^ Genome Analysis ToolKit (version 3.7) was used for realignment.^23^ Finally, the processed BAM file was obtained and used for the subsequent analyses.

VarDict (version 1.5.1) was used to call single nucleotide variant (SNV),^24^ and compound heterozygous mutations were merged using FreeBayes (version 1.2.0).^25^ Tumor‐normal paired sample calling was performed during the mutation‐calling procedure to filter out germline mutations. After annotating with ANNOVAR, somatic mutations were selected based on the following criteria: (i) located in intergenic regions or intronic regions, (ii) synonymous SNVs, (iii) allele frequency ≥0.002 in the Exome Aggregation Consortium and Genome Aggregation Database, (iv) allele frequency <0.05 in the tumor sample and allele frequency <0.05 in the normal sample, (v) strand bias mutations in the reads, (vi) support reads <5, and (vii) depth < 120. We identified copy number variations (CNV) in the tumor samples compared to normal samples using CNVkit software (version 0.9.2).^26^ The threshold set for identifying CNV gain was a copy number of 3, and 1.2 for CNV loss. Variant allele frequencies (VAF) were computed as the ratio of alternate allele counts to the total read depths at each genomic position. We adapted the mutant allele tumor heterogeneity (MATH) score to encompass all somatic variants having a VAF ranging from 0.02 to 1. This modification calculated the score as 100 times the median absolute deviation divided by the median VAF. The tumor mutational burden (TMB) of the tumor samples was determined by first ascertaining the absolute mutation counts of the tumor samples relative to the mutation spots in the normal samples. This calculation was done using the formula: Absolute mutation counts × 1,000,000/panel exonic base num. TMB was measured as the number of mutations per Mb.

Synonymous and nonsynonymous somatic SNVs were investigated to discern the patterns of mutational signatures. Six primary categories of base substitutions, T > A, T > C, T > G, C > A, C > G, and C > T, were considered across all samples. Given the nucleotides flanking the mutated base at the 5′ and 3′ ends, 96 distinct substitution classifications were identified. The underlying mutational signatures were extracted using the R package ‘mutational patterns’ and nonnegative matrix factorization. The differential contributions of these signatures in patients categorized as LN‐negative and LN‐positive were examined. Finally, the derived signatures were cross‐referenced with established COSMIC signatures available at (http://cancer.sanger.ac.uk/cosmic/signatures).

The Genomic Identification of Significant Targets in Cancer (GISTIC) algorithm is utilized to pinpoint regions of genomic variation that are more likely to be involved in cancer development.^27^ Genomic regions that exhibit amplification and deletion across numerous samples can be visualized. In the Ruijin cohort, patients with or without LN were analyzed using GISTIC 2.0. Differences in copy numbers between the two groups were compared.

### 2.3 Construction and benchmark of the predictive model

Eight machine learning algorithms were integrated to establish a consensus‐predictive model exhibiting robust accuracy and stability. The ensemble of algorithms included support vector machine (SVM), N‐NET, GLM, LASSO, K‐Nearest Neighbor (KNN), LR, gradient boosting machine (GBM), and random forest (RF). The procedure for signature generation was delineated as follows: (a) significant variates in the Ruijin cohort were determined using univariate Cox regression; (b) prediction models were fitted to these significant variates using nine algorithmic models within a 5‐fold cross‐validation framework; (c) these models were evaluated in the HS validation datasets; and (d) the accuracy of each model was determined, and ROC analysis was executed across all validation datasets. The model with the highest AUC was considered optimal.

### 2.4 Statistical analysis

The Chi‐square test and Fisher exact test was used to compare categorical variables, while the Mann–Whitney test was used for analyzing nonparametric variables. Fisher's exact test was utilized to assess the frequencies of gene alterations that occurred in 2% or more of the entire study cohort. Additionally, we calculated the significance of co‐occurrence or mutual exclusivity of each gene of interest using a pairwise Fisher exact test. Univariate and multivariate logistic analysis were used to screen factors related to LN metastasis. A two‐tailed *p* < 0.05 was considered statistically significant. Statistical analysis was performed using GraphPad Prism 9.0.2 (GraphPad Software, MA, USA) or R 4.1.3 (R Core Team, Austria).

## 3 RESULTS

### 3.1 Clinicopathologic features of pT1 patients with and without LN

A total of 224 patients in Ruijin hospital and 140 patients in Huashan hospital were included in this study, among them, 24 (10.7%) and 14 (10.0%) patients had LN metastases, respectively. The clinicopathologic features of these two cohorts are summarized in Table 1. No differences were observed in age and tumor location between the LN‐positive and LN‐negative groups in both cohorts. On CT imaging review, LN‐positive tumor showed a lower rate of subsolid morphology in both Ruijin cohort (20.83% vs. 68.00%, *p* < 0.001) and Huashan cohort (14.29% vs. 66.67%, *p* < 0.001). On pathologic review, the presence of intravascular tumor emboli (both *p* < 0.001) and micropapillary component (*p* = 0.002, *p* = 0.010, respectively) was detected in LN‐positive tumors in both Ruijin cohort and Huashan cohort. Moreover, LN‐positive patients trended to have a smoking history (*p* = 0.012, *p* = 0.187, respectively) and higher pathological T stage (*p* = 0.011, *p* = 0.179, respectively) in both Ruijin cohort and Huashan cohort.

**TABLE 1:** Clinicopathologic features of T1 LUAD patients across two cohorts.

Characteristic	Train (Ruijin cohort)			Test (Huashan cohort)		
	LN‐negative	LN‐positive	*p* Value	LN‐negative	LN‐positive	*p* Value
Number	200	24		126	14	
Sex						
Male	73 (36.50%)	15 (62.50%)	0.014	56 (44.44%)	4 (28.57%)	0.255
Female	127 (63.50%)	9 (37.50%)		70 (55.56%)	10 (71.43%)	
Age	60 (53.68)	61 (50.67)	0.621	62 (54.69)	67 (61.69)	0.278
Tumor location						
RUL	66 (33.00%)	8 (33.33%)	0.061	38 (30.16%)	6 (42.86%)	0.444
RML	17 (8.50%)	1 (4.17%)		9 (7.14%)	1 (7.14%)	
RLL	42 (21.00%)	6 (25.00%)		23 (18.25%)	4 (28.57%)	
LUL	53 (26.50%)	2 (8.33%)		37 (29.37%)	3 (21.43%)	
LLL	22 (11.00%)	7 (29.17%)		19 (15.08%)	0	
Smoking status						
No	149 (74.50%)	12 (50.00%)	0.012	93 (73.81%)	8 (57.14%)	0.187
Yes	51 (25.50%)	12 (50.00%)		33 (26.19%)	6 (42.85%)	
Morphology on CT						
Pure GGO	10 (5.00%)	0	<0.001	9 (7.14%)	0	0.001
Mixed GGO	126 (63.00%)	5 (20.83%)		73 (57.94%)	2 (14.29%)	
Solid	64 (32.00%)	19 (79.17%)		44 (34.92%)	12 (85.71%)	
Subsolid tumor on CT						
No	64 (32.00%)	19 (79.17%)	<0.001	42 (33.33%)	12 (85.71%)	<0.001
Yes	136 (68.00%)	5 (20.83%)		84 (66.67%)	2 (14.29%)	
pT stage						
pT1a	47 (23.50%)	2 (8.33%)	0.011	11 (8.73%)	0	0.179
pT1b	113 (56.50%)	11 (45.83%)		79 (62.70%)	7 (50.00%)	
pT1c	40 (20.00%)	11 (45.83%)		36 (28.57%)	7 (50.00%)	
Tumor differentiation						
G1	2 (1.00%)	0	0.363	1 (0.79%)	0	0.023
G1‐2	23 (11.50%)	1 (4.17%)		0	0	
G2	105 (52.50%)	9 (37.50%)		4 (3.17%)	0	
G2‐3	36 (18.00%)	7 (29.17%)		0	0	
G3	6 (3.00%)	1 (4.17%)		0	1 (7.14%)	
Unkown	28 (14.00%)	6 (25.00%)		121 (96.03%)	13 (92.86%)	
Intravascular tumor thrombus						
No	186 (93.00%)	12 (50.00%)	<0.001	122 (96.83%)	10 (71.43%)	<0.001
Yes	6 (3.00%)	8 (33.33%)		3 (2.38%)	4 (28.57%)	
Unkown	8 (4.00%)	4 (16.67%)		1 (0.79%)	0	
Micropapillary component						
No	178 (89.00%)	16 (66.67%)	0.002	88 (69.84%)	5 (35.71%)	0.010
Yes	22 (11.00%)	8 (33.33%)		38 (30.16%)	9 (64.29%)	
Solid component						
No	170 (85.00%)	17 (70.83%)	0.077	8 (6.35%)	0	0.332
Yes	30 (15.00%)	7 (29.17%)		118 (93.65%)	14 (100.00%)	
PD‐L1 TPS						
<1%	86 (43.00%)	8 (33.33%)	0.066	62 (49.21%)	5 (35.71%)	0.531
≥1%	35 (17.50%)	9 (37.50%)		62 (49.21%)	9 (64.29%)	
Unkown	79 (39.50%)	7 (29.17%)		2 (1.59%)	0	

*Note*: Data are presented as no. (%) or median (interquartile range).

Abbreviations: CT, computed tomography; GGO, ground‐glass opacity; LLL, left lower lobe; LUL, left upper lobe; RLL, right lower lobe; RML right middle lobe; RUL right upper lobe.

### 3.2 Genomic landscape of pT1 LUAD

**FIGURE 1:**
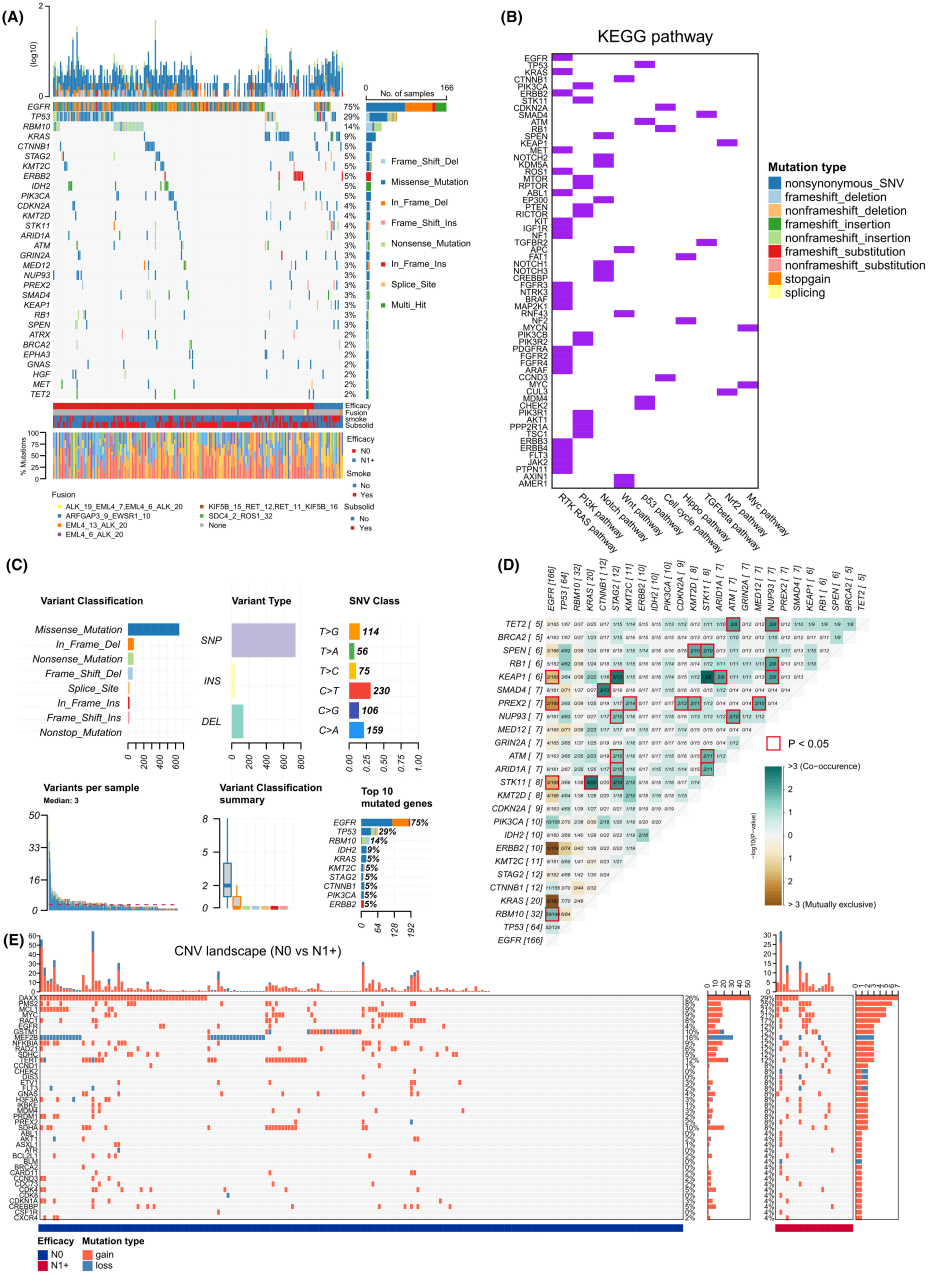
Somatic mutations in T1 LUAD patients. (A) Oncoprint displaying the top 20 genes altered in patients diagnosed with T1 LUAD in Ruijin cohort. (B) Pathway analysis of altered genes in T1 LUAD tumors. (C) SNV subtypes of somatic gene alternations. (D) Patterns of cooccurring and mutually exclusive gene alterations. (E) CNV oncoprint of T1 LUAD. CNV, copy number variation; LUAD, lung adenocarcinoma; KEGG, Kyoto Encyclopedia of Genes and Genomes; N0, LN‐negative; N1+, LN‐positive.

To investigate the molecular characteristics of patients with LN‐positive and LN‐negative tumors, we enriched and analyzed their genomic DNA. The top 20 gene alternations, including SNV and efficacy fusion, are shown in Figure 1A. *EGFR* mutation, the most frequent driver gene mutation in LUAD, was detected in 78% (175/224) of the patients with T1 LUAD, followed by *TP53* mutations (29%, 65/224) and *RBM10* mutations (15%, 34/224). These gene alterations were enriched in RTK/RAS pathway, PI3K pathway, Notch pathway, etc. (Figure 1B). SNV subtypes of somatic gene alternations was shown in Figure 1C. Patterns of the co‐occurrence of gene alterations and mutual exclusivity were observed among patients with pT1 LUAD in the study cohort (Figure 1D). In total, 26 gene pairs were co‐altered at a statistically significant frequency, and five pairs were mutually exclusive. Notably, genes, including *KEAP1*, *PREX2*, *STK11*, *ERBB2*, and *KRAS*, with mutually exclusive mutations only occurred in patients with *EGFR* mutant tumors. The top three most frequent CNV in patients with pT1 LUAD were *DAXX*, *TERT* amplification and *MEF2B* loss (Figure 1E).

### 3.3 Analysis of MATH, TMB, TNB, and CNI in LN‐positive and LN‐negative T1 LUAD tumors

**FIGURE 2:**
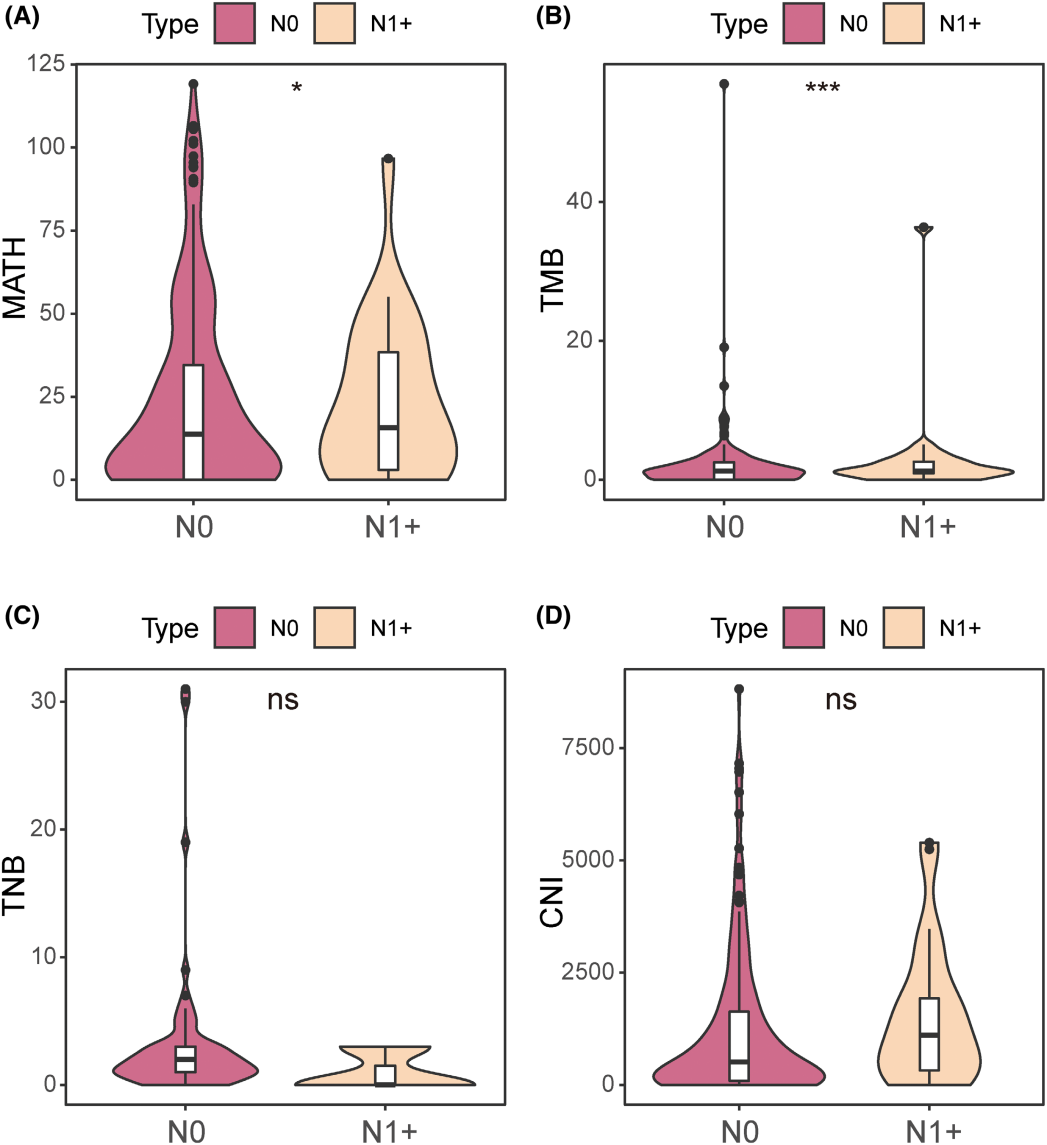
Analysis of MATH, TMB, TNB and CNI in LN‐positive and LN‐negative T1 LUAD tumors. (A) Comparison of MATH between the two groups of patients. (B) Comparison of TMB between the two groups of patients. (C) Comparison of tumor neoantigen burden between the two groups of patients. (D) Comparison of CNI between the two groups of patients. CNI, copy number instability; MATH, mutant allele tumor heterogeneity; ns, not significant; N0, LN‐negative. N1+, LN‐positive; TMB, tumor mutation burden; TNB, tumor neoantigen burden; *p < 0.05, ***p < 0.001.

We then compared the MATH, TMB, tumor neoantigen burden (TNB), and copy number instability (CNI) scores between the patients with LN‐positive and LN‐negative tumors. The MATH score (*p* = 0.02916, median value = 14.315, 0 ~ 119.14) (Figure 2A) and TMB score (*p* = 0.00032, median value = 1.271, 0 ~ 56.9778) (Figure 2B) of the LN‐positive group were significantly higher than that of LN‐negative group. And the increasing trend of TNB (*p* = 0.054, median value = 2, 0 ~ 31) (Figure 2C) and CNI (*p* = 0.12392, median value = 548.44, 0 ~ 8813.36) (Figure 2D) was observed in LN‐positive group.

### 3.4 Comparison of gene mutation characteristics between tumors with or without LN metastases

**FIGURE 3:**
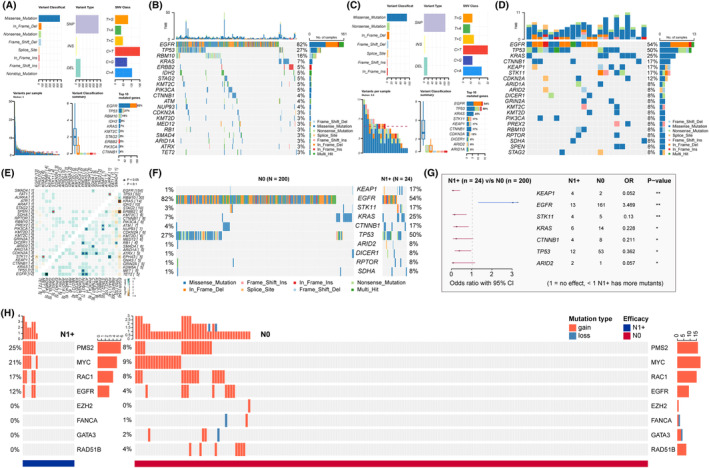
Comparison of gene mutation characteristics between tumors with or without lymph node metastases. (A) SNV subtypes of somatic gene alternations in patients with LN‐negative tumors. (B) Oncoprint displaying the top 20 genes altered in patients with LN‐negative tumors. (C) SNV subtypes of somatic gene alternations in patients with LN‐positive tumors. (D) Oncoprint displaying the top 20 genes altered in patients with LN‐positive tumors. (E) Patterns of co‐occurring (green block) and mutually exclusive (brown block) gene alterations in LN‐negative (bottom right) and LN‐positive tumors (top‐left). (F) Oncoprint displaying the top 10 genes altered between patients with LN‐positive tumors and those with LN‐negative tumors. (G) Genes altered at significantly different frequencies between the two groups of patients determined using univariable analysis. (H) Significant copy number alternations between the two groups of patients. LN, lymph node; N0, LN‐negative; N1+, LN‐positive; SNV, single nucleotide variant.*p < 0.05, **p < 0.01.

Next, we analyzed the SNV subtypes (Figure 3A,C) and constructed an oncoprint (Figure 3B,D) of the patients with LN‐positive or LN‐negative tumors. The top 10 differential genes are shown in Figure 3F. *EGFR* mutations were observed in 80% of the patients with LN‐negative tumors and 54% with LN‐positive tumors (*p* < 0.01). *KEAP1* (*p* < 0.01), *STK11* (*p* < 0.01), *KRAS* (*p* < 0.05), *CTNNB1* (*p* < 0.05), *TP53* (*p* < 0.05), and *ARID2* (*p* < 0.05) mutation were increased in the patients with LN‐positive tumors (Figure 3G). Unique comutation of *KRAS*/*STK11* (also called *LKB1*) and *SPEN*/*STK11* were observed in LN‐positive tumors (top‐left, Figure 3E). We further examined the association between CNV and pathological LN metastasis. As shown in Figure 3H, higher *PMS2* (N1+ 25% vs. N0 8%), *MYC* (N1+ 21% vs. N0 9%), *RAC1* (N1+ 17% vs. N0 8%), and *EGFR* (N1+ 12% vs. N0 4%) amplifications were observed in the patients with LN‐positive tumors than in the patients with LN‐negative tumors. GISTIC plots of focal copy number gains and losses across the LN‐positive and LN‐negative cohorts are shown in Figure S1.

Subsequently, we analyzed the mutation spectra of LN‐positive and LN‐negative patients and analyzed mutation signatures according to the COSMIC database. After NMF clustering, patients without LN were divided into eight signatures, and patients with LN were divided into six signatures. Among LN‐negative patients, we found a relatively high prevalence of mutations mediated by APOBEC enzymes and mutations related to DNA mismatch repair (MMR) deficiency (Figure S2A), whereas, in LN‐positive patients, a relatively high occurrence of mutations associated with anticancer drug resistance and smoking (Figure S2B) was observed. Additionally, LN‐positive patients also had a relatively low cosine similarity, which may be related to the small sample size of these patients. Furthermore, we noted that both LN‐negative patients and LN‐positive patients exhibited increased copy number amplifications and deletions, such as those in 1p11.2, 1q23.1, and 1q23.3. Compared with LN‐negative patients, LN‐positive patients showed significant copy number amplifications at 17q24.2 and significant copy number deletions at 19p13.12. These structural mutations could be used as molecular markers.

### 3.5 Model construction and benchmark testing for predicting LN metastasis

**FIGURE 4:**
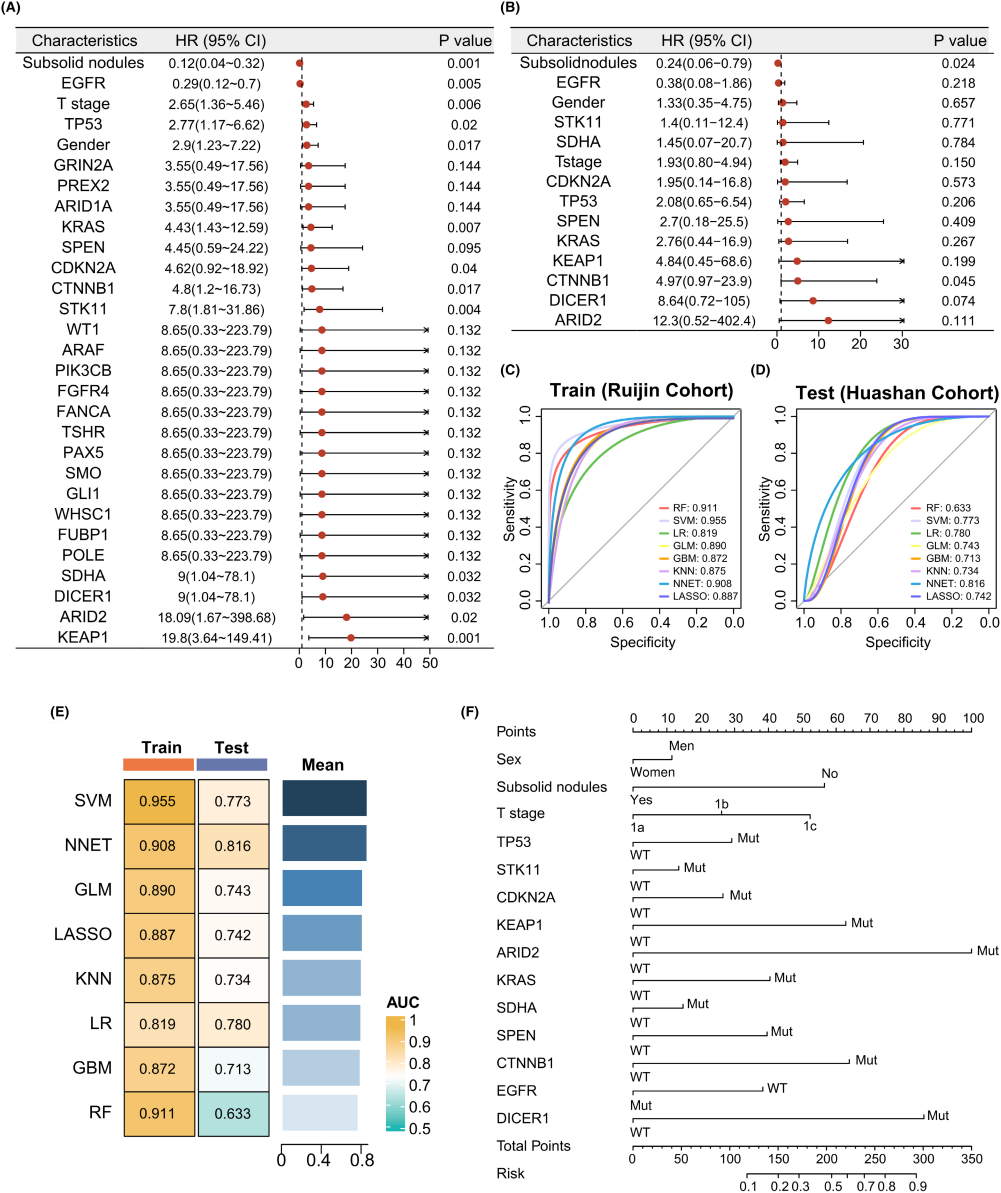
Model construction and benchmark testing for predicting lymph node metastasis. (A) Univariate logistic analysis used to screen factors related to lymph node metastasis revealed 29 variables. (B) Multivariate logistic stepwise regression used to select variables presented 14 variables after removing collinearity. (C) Benchmark testing in the training set using eight machine learning algorithms, ROC curve plot, and model accuracy ranking based on AUC values, from the highest to the lowest: SVM, RF, NNET, GLM, LASSO, KNN, GBM, and LR. (D) Benchmark testing in the test set using eight machine learning algorithms, ROC curve plot, and model accuracy ranking based on AUC values, from the highest to the lowest: NNET, LR, SVM, GLM, KNN, LASSO, GBM, and RF. (E) AUC values in the training and test sets, along with the average AUC values. (F) Lymph node metastasis prediction nomogram based on logistic regression. AUC, area under the curve; GBM, gradient boosting machine; KNN, K‐Nearest Neighbor; LN, lymph node; N0, LN‐negative; N1+, LN‐positive; NNET, neural network; RF, random forest; ROC, receiver operating characteristic; SVM, support vector machine.

The univariate logistic analysis identified 29 variables associated with LN metastasis based on the clinical characteristics and mutation data from the Ruijin cohort (Figure 4A). To eliminate collinear variables in the model, we performed the multivariate logistic analysis using stepwise regression (direction = “both”) on the 29 variables and constructed a predictive model (Figure 4B). To determine the optimal model, we used the Ruijin and Huashan cohorts as the training and test sets, respectively. We fitted eight predictive models through 5‐fold cross‐validation and plotted receiver operating characteristic (ROC) curves for the training and test sets (Figure 4C,D). Furthermore, we calculated each model's accuracy, precision, recall, and F1 scores in the test set (Table 2). The area under the curve (AUC) results indicated that the SVM model exhibited the best predictive performance, followed by N‐NET, GLM, LASSO, KNN, LR, GBM, and RF (Figure 4E). While LR ranked sixth in AUC on the training set, it performed second best on the test set (AUC = 0.780, test cohort). Compared to other models, LR offers better interpretability; therefore, we constructed a column chart including sex, subsolid morphology, T stage, and 11 genes mutation status based on LR for a more intuitive assessment of patient risk scores (Figure 4F). The visualized genetic mutation data for the 11 genes involved are shown in Figure S3.

**TABLE 2:** Evaluation metrics for each model in the test set.

Model	Accuracy	Precision	Recall	F1 score	Kappa
GLM	0.871	0.961	0.904	0.931	−0.054
LASSO	0.871	0.961	0.904	0.931	−0.054
NNET	0.871	0.953	0.91	0.931	0.037
RF	0.907	1	0.907	0.951	0
KNN	0.907	1	0.907	0.951	0
SVM	0.85	0.929	0.908	0.918	0.007
LR	0.843	0.921	0.907	0.914	−0.002
GBM	0.879	0.969	0.904	0.935	−0.046

## 4 DISCUSSION

Given the paramount importance of LN staging for the therapeutic strategies in lung cancer patients, preoperative detection of LN metastases could facilitate the matching of patients into an approximate surgical type. Routine examinations are either invasive (e.g., endobronchial ultrasound bronchoscopy and mediastinoscopy) or have unsatisfactory detection efficacy (e.g., PET‐CT). Here, a comprehensive analysis was conducted on the genomic profiling and clinicopathologic characteristics of patients with T1 LUAD to fathom the distinctions between early invasive LUADs with or without LN metastases. Subsequently, a prediction model was established and validated.

In the entire cohort of patients, the observed probability of LN metastasis was 10.44% (38/364). Our findings revealed that, among the various clinicopathological features examined, tumors exhibiting LN metastases were characterized by solid nodules on CT imaging, larger nodule diameters, the presence of intravascular tumor emboli, and micropapillary components within the tumor mass. In accordance with the reported data,^28^ our study observed that the probability of LN metastasis in cases of pure ground‐glass opacity (GGO) was zero (0/19). The probability of LN metastasis was 3.40% (7/206) in mixed GGOs and significantly higher at 22.30% (31/139) in solid nodules. For tumors with intravascular tumor emboli and micropapillary components, the probability of LN metastasis was 57.14% (12/21) and 22.08% (17/77), respectively. Micropapillary components, even in minor components, were found to be associated with LN metastases, early recurrence, and poor prognosis.^7^, ^29^ Here, we found this kind of relevance in T1 tumors.

In 1890, David von Hansemann elucidated the first insights about the pivotal role of genetic alterations in cancer development.^30^ Cancer is the dysregulated proliferation of cells caused by key driver gene mutations, and its development is a consequence of a complex interplay between specific mutated and nonmutated driver genes. These genes orchestrate tumorigenesis by modulating oncogenic pathways to an optimal degree, thereby facilitating tumor growth and evolution. Thus, different mutational landscapes may represent different tumor characteristics. Several recent studies have already demonstrated that different genetic features in NSCLC lead to different propensities for LN metastases.^18^, ^19^, ^31^, ^32^ In the present study, we analyzed the genetic characteristics of early invasive LUADs, focusing on group comparisons. Our findings revealed that in comparison to tumors without LN metastasis, tumors with LN metastasis were more likely to exhibit *EGFR* wild‐type and/or mutations in genes such as *KEAP1*, *STK11*, *KRAS*, *CTNNB1*, *TP53*, and *ARID2*. NSCLC patients harboring the *EGFR* mutation exhibit distinct clinical characteristics, such as adenocarcinoma, female gender, never smokers, and Asian ethnicity.^33^ However, its relevance to LN metastasis remains controversial.^18^, ^19^, ^34^, ^35^, ^36^, ^37^, ^38^ Among these studies, an interesting geographic disparity was observed. Consistent with our study, patients with *EGFR* mutations in East Asian countries exhibited lower rates of LN metastasis.^18^, ^34^, ^35^, ^36^ Conversely, in West Asian and European countries, patients with EGFR mutations showed a higher or similar likelihood of LN metastasis.^19^, ^37^, ^38^ This phenomenon may be attributed to the interplay between driver mutant genes and other genetic factors and also underscores the variations in genetic profiles across human races. We examined patterns of co‐occurring and mutually exclusive gene alterations to advance the understanding of specific driver gene mutation patterns across T1 LUAD genomes. We found the unique comutation of *KRAS*/*STK11* (also known as *LKB1*) and *SPEN*/*STK11* in LN‐positive tumors. *STK11* functions as the principal upstream activator of AMPK, an energy sensor that is activated under conditions of low ATP concentrations, leading to the inhibition of mTORC1. Consequently, in the absence of *STK11*, lung cancers harboring *KRAS* mutations not only gain a growth advantage due to the unrestrained signaling of mTOR but also exhibit mitochondrial dysfunction,^39^ resulting in aggressive behavior. Loss of *Lkb1* in *Kras*‐driven lung tumors results in a higher LN metastase rate in mice.^40^ In clinic, *KRAS*/*STK11* comutation patients showed a poorer prognosis and primary resistance to PD‐1 axis inhibitors.^41^, ^42^ As far as we know, this is the first study indicating a higher possibility of LN metastasis in early LUAD with comutation of *KRAS*/*STK11 or SPEN*/*STK11*. Differences in MATH and TMB were detected between the patients with or without LN metastasis. However, these two parameters were not incorporated into the final model. The model was designed to include only metrics that are readily accessible preoperatively, to facilitate the ease of use for validation of the model in the future.

While a similar study has explored the clinicopathologic and genomic differences in patients with and without LN metastases,^19^ our study is distinct as it focuses on an East Asian population. As previously mentioned, the East Asian population exhibits significant genomic differences from other regions. Additionally, our research specifically targets T1‐stage LUAD. This stage of lung cancer constitutes a substantial proportion of clinical cases, accounting for 45.2% to 56.0% in recently published studies^43^, ^44^ and 70.9% in our center (unpublished data). Furthermore, the precision of LN staging assumes a greater significance in early‐stage lung cancer. Another strength of our study is that we validated our model wtih another separate cohort. Other studies use artificial intelligence to analyze histopathologic images^45^, ^46^ or radiomics^47^, ^48^ to predict LN metastasis in lung cancer. In the future, the application of artificial intelligence combined with multi‐omics may be able to predict LN metastasis of early lung cancer more accurately.

This study has some limitations. First, the small number of patients in the LN‐positive group may have led to statistical bias. Second, our model was not validated using preoperative specimens. Obtaining specimens preoperatively may be challenging, but recent studies have shown that detection rates of actionable genomic biomarkers are similar between NGS‐based ctDNA assays and tissue‐based methods.^49^, ^50^ Moreover, small tumor tissue samples obtained from CT‐guided needle biopsy or bronchoscopy also have a relatively high success rate in obtaining high‐quality DNA.^51^

In conclusion, we performed an integrative analysis of the clinicopathological and genomic variables in a large cohort of patients with T1 LUAD. The findings highlight the potential importance of genomic data in identifying patients at risk of pathologic LN metastasis. Our model could identify patients with T1 LUAD who are at a high risk of pathological LN metastasis, thereby potentially guiding therapeutic strategies before surgical resection.

## AUTHOR CONTRIBUTIONS

**Wei Guo:** Conceptualization (lead); data curation (lead); formal analysis (lead); investigation (lead); methodology (lead); writing – original draft (lead). **Tong Lu:** Data curation (lead); formal analysis (equal); investigation (equal); methodology (equal); writing – original draft (equal). **Yang Song:** Data curation (equal); formal analysis (lead); investigation (equal); methodology (equal); writing – original draft (equal). **Anqi Li:** Data curation (equal); formal analysis (equal); investigation (equal); methodology (lead); writing – original draft (equal). **Xijia Feng:** Data curation (equal); formal analysis (equal); investigation (lead); methodology (equal); writing – original draft (equal). **Dingpei Han:** Methodology (equal); resources (equal). **Yuqin Cao:** Formal analysis (equal). **Debin Sun:** Formal analysis (equal). **Xiaoli Gong:** Formal analysis (equal). **Chengqiang Li:** Resources (equal). **Runsen Jin:** Resources (equal). **Hailei Du:** Resources (equal). **Kai Chen:** Resources (equal). **Jie Xiang:** Resources (equal). **Junbiao Hang:** Resources (equal). **Gang Chen:** Project administration (lead); resources (lead); supervision (lead); writing – review and editing (lead). **Hecheng Li:** Project administration (lead); resources (lead); supervision (lead); writing – review and editing (lead).

## CONFLICT OF INTEREST STATEMENT

Debin Sun and Xiaoli Gong were employed by company Genecast Biotechnology Co., Ltd. Other authors declare that they have no conflict of interest.

## ETHICS STATEMENT

All processes strictly adhered to the guidelines of the Ethics Committee and were in accordance with the principles of the Declaration of Helsinki. This study was approved by the Ethics Committees of Ruijin Hospital and Huashan Hospital.

## Supporting information


Figure S1.

Figure S2.

Figure S3.

Table S1.

## Data Availability

Genomic and clinical data are available from the corresponding authors upon reasonable request.
