# Supplementary figures and images for: Lymph node metastasis in early invasive lung adenocarcinoma: Prediction model establishment and validation based on genomic profiling and clinicopathologic characteristics
Source: Cancer Med. 2024 Jul 24;13(14):e70039. doi: 10.1002/cam4.70039 (PMC11267562; doi:10.1002/cam4.70039)

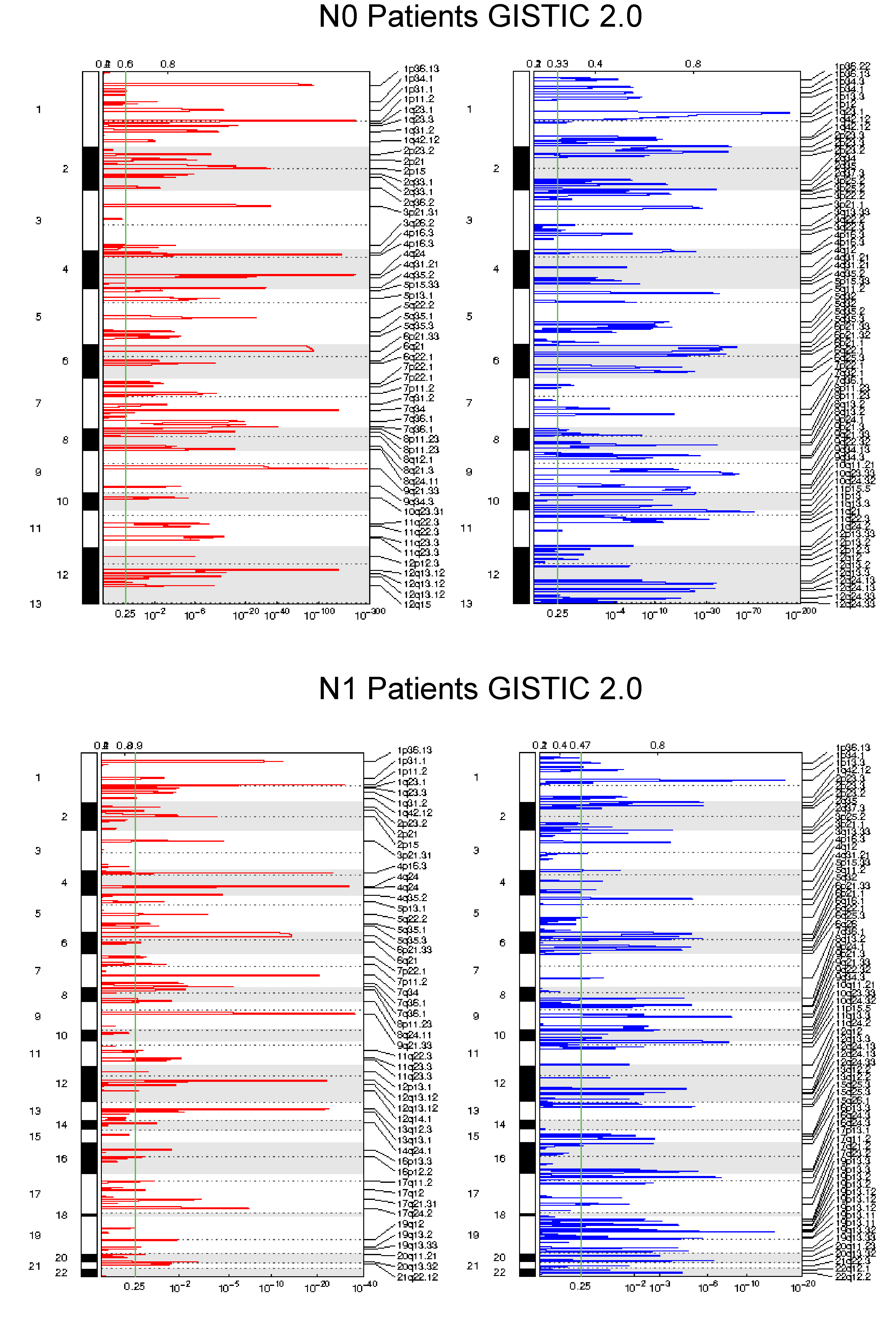

Supplement: Supplementary file 1 — Figure S1. [file CAM4-13-e70039-s003.tif]

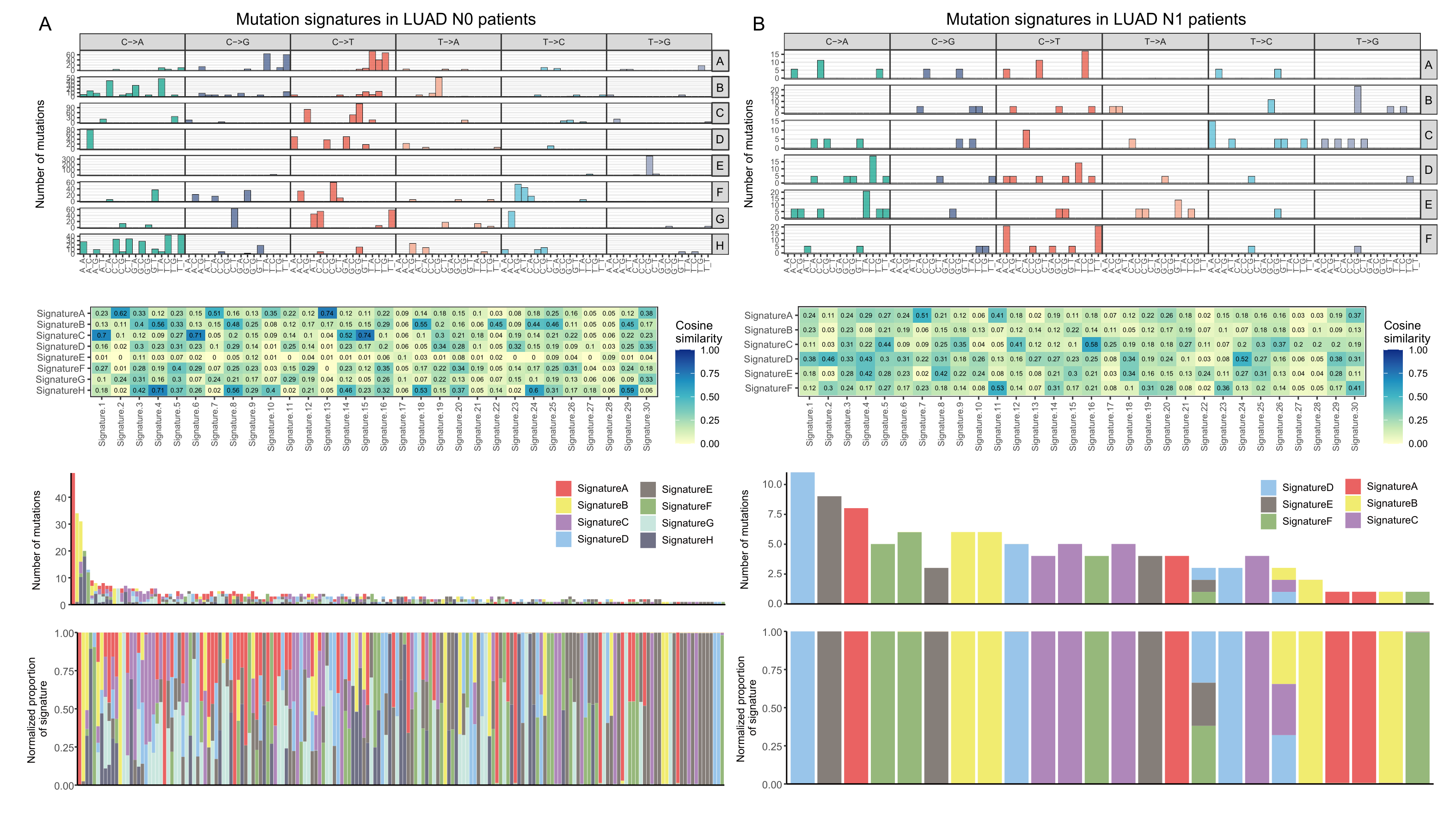

Supplement: Supplementary file 2 — Figure S2. [file CAM4-13-e70039-s002.tiff]

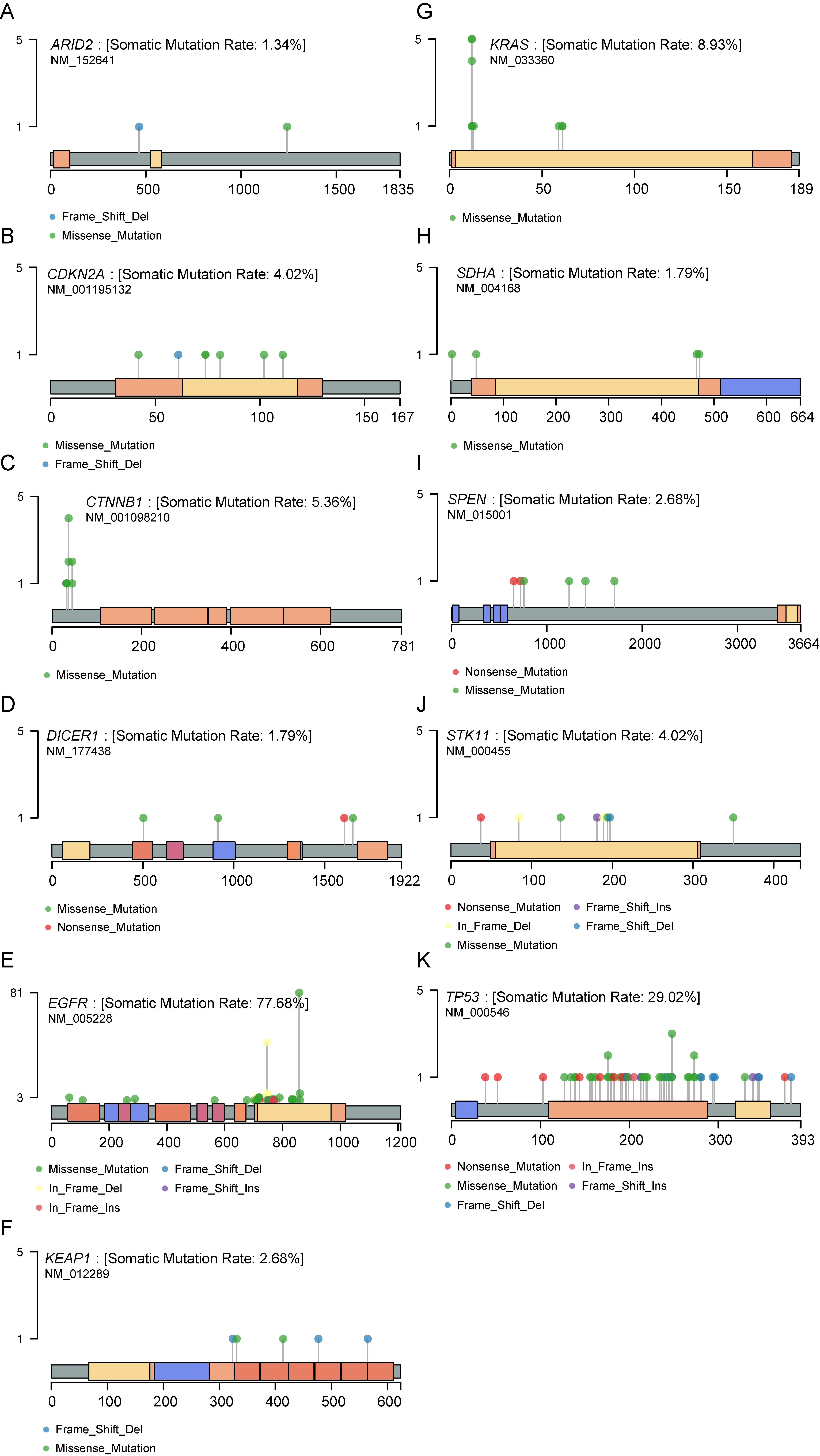

Supplement: Supplementary file 3 — Figure S3. [file CAM4-13-e70039-s001.tif]
