# Supplementary material for: Lymph node metastasis in early invasive lung adenocarcinoma: Prediction model establishment and validation based on genomic profiling and clinicopathologic characteristics
Source: Cancer Med. 2024 Jul 24;13(14):e70039. doi: 10.1002/cam4.70039 (PMC11267562; doi:10.1002/cam4.70039)
Supplement: Supplementary file 4 — Table S1. [file CAM4-13-e70039-s004.docx]

Table S1. Gene list

| ABCA13 | ABCA8 | ABCB1 | ABCC2 | ABCC9 | ABL1 |
| --- | --- | --- | --- | --- | --- |
| ACADSB | ACOT13 | GCNA | ADCY8 | ADGRG6 | AGAP1 |
| AK7 | AKT1 | AKT2 | AKT3 | ALDH5A1 | ALG9 |
| ALK | ALOX12B | C2CD6 | AMBRA1 | AMER1 | ANAPC7 |
| ANKRD28 | ANKRD46 | ANO1 | APAF1 | APC | APOL2 |
| APOPT1 | AQR | AR | ARAF | ARHGAP26 | ARHGAP4 |
| ARHGAP6 | ARHGEF12 | ARHGEF3 | ARID1A | ARID1B | ARID2 |
| ARID4A | ARID5B | ARL13B | ARL4A | ARL6IP6 | ARMC5 |
| ASB11 | ASH1L | ASPH | ASXL1 | ASXL2 | ATG3 |
| ATG4C | ATIC | ATM | ATP6V0A1 | ATP6V0A2 | ATP6V0A4 |
| ATP6V0E1 | ATP8A1 | ATR | ATRX | AURKA | AURKB |
| AXIN1 | AXIN2 | AXL | B2M | BAP1 | BARD1 |
| BCAS1 | BCL2 | BCL2L1 | BCL6 | BCOR | BIRC3 |
| BLM | BMPR1A | BRAF | BRCA1 | BRCA2 | BRD4 |
| BRIP1 | BRMS1L | BRS3 | BTF3 | BTG1 | BTK |
| C22orf23 | C5orf15 | CPLANE1 | C7orf66 | C8orf34 | CAB39 |
| CACNA1E | CACNA2D1 | CALD1 | CALM2 | CALR | CARD11 |
| CASP8 | CAST | CBFB | CBL | CBR3 | CBR4 |
| CCDC157 | CCDC18 | CCND1 | CCND2 | CCND3 | CCNE1 |
| CD274 | CD40 | CD74 | CD79A | CD79B | CDA |
| CDC73 | CDCA8 | CDH1 | CDK12 | CDK4 | CDK6 |
| CDK8 | CDKL3 | CDKN1A | CDKN1B | CDKN2A | CDKN2B |
| CDKN2C | CDO1 | CEBPA | CEP120 | CEP290 | CFAP221 |
| CFAP53 | CHD1 | CHEK1 | CHEK2 | CIC | CLASP2 |
| CLEC16A | CLEC9A | CNKSR3 | CNOT8 | COL15A1 | COX18 |
| CPS1 | CREBBP | CRKL | CRLF2 | CSF1R | CSF3R |
| CTAGE5 | CTCF | CTLA4 | CTNNB1 | CTSC | CUL3 |
| CXCL8 | CXCR4 | CYBA | CYFIP1 | CYLD | CYP19A1 |
| CYP2B6 | CYP2C19 | CYP2C8 | CYP2D6 | DARS2 | DAXX |
| DCHS2 | DDR1 | DDR2 | DDX19B | DDX58 | DEPDC5 |
| DHFR | DIAPH1 | DIAPH2 | DICER1 | DIS3 | DLC1 |
| DMXL1 | DNAJC11 | DNMT1 | DNMT3A | DNMT3B | DOCK11 |
| DOT1L | DPP6 | DPYD | DSCAM | E2F3 | EBP |
| EED | EGFR | EIF1AX | EIF4E | EIF4G3 | ELFN1 |
| ELMOD2 | EML4 | ENOSF1 | ENSA | EP300 | EPCAM |
| EPG5 | EPHA3 | EPHA5 | EPHA7 | EPHB1 | EPYC |
| ERBB2 | ERBB3 | ERBB4 | ERCC1 | ERCC2 | ERCC3 |
| ERCC4 | ERG | ERI1 | ERRFI1 | ESR1 | ETV1 |
| ETV4 | ETV5 | ETV6 | EWSR1 | EXOSC8 | EZH2 |
| EZR | FAM149A | FAM153B | FAM161A | FAM175A | FAM184B |
| TENT5C | FANCA | FANCC | FANCD2 | FANCF | FANCG |
| FAS | FAT1 | FBXO11 | FBXW7 | FGF16 | FGF19 |
| FGF3 | FGF4 | FGFR1 | FGFR2 | FGFR3 | FGFR4 |
| FH | FLCN | FLOT1 | FLT1 | FLT3 | FLT4 |
| FMNL2 | FMO1 | FMR1 | FNBP4 | FOLH1B | FOXA1 |
| FOXL2 | FOXO1 | FOXP1 | FUBP1 | FUS | FXR1 |
| GABRP | GALNT12 | GALNT14 | GANC | GATA1 | GATA2 |
| GATA3 | GIPC1 | GLI1 | GMEB1 | GNA11 | GNA13 |
| GNAQ | GNAS | GPAT3 | GPC4 | GPM6A | GRB10 |
| GREM1 | GRIK2 | GRIN2A | GSK3B | GSKIP | GSTA1 |
| GSTM1 | GSTP1 | GUCY1A2 | H3F3A | HAUS2 | HAUS6 |
| HCAR2 | HDGFL3 | HERC6 | HEY1 | HGF | H1-2 |
| H3C2 | HLA-A | HLA-B | HLA-C | HMCN1 | HNF1A |
| HNF4A | HOMER1 | HRAS | HSD17B11 | HSD3B1 | HSPA1B |
| HSPA4 | HSPA5 | HSPH1 | HTT | HYOU1 | IARS |
| ICOSLG | ID2 | ID3 | IDH1 | IDH2 | IGF1 |
| IGF1R | IGF2 | IKBKE | IKZF1 | IL10 | IL13RA1 |
| IL7R | IMPG1 | INHBA | INPP4A | INPP4B | IRF4 |
| IRF6 | IRF8 | IRS2 | ITGAL | JAK1 | JAK2 |
| JAK3 | JUN | KDM5A | KDM5C | KDM6A | KDR |
| KEAP1 | KIAA1210 | KIAA1841 | KIF5B | KIT | KLF4 |
| KMT2A | KMT2C | KMT2D | KPNA4 | KPNB1 | KRAS |
| KTN1 | LAMA3 | LATS1 | LATS2 | LEPR | LMO1 |
| LNPEP | LONRF3 | LRP2 | CARMIL1 | LRRC34 | LYN |
| MALRD1 | MALT1 | MAP2K1 | MAP2K2 | MAP2K4 | MAP3K1 |
| MAP3K13 | MAP3K4 | MAP4K3 | MAP4K5 | MAPK1 | MAPKAP1 |
| MAPKBP1 | MARK1 | MARK3 | MAX | MCL1 | MDC1 |
| MDM2 | MDM4 | MED12 | MED12L | MED14 | MED19 |
| MEF2B | MEIS1 | MEN1 | MET | METTL9 | MITF |
| MLH1 | MLH3 | MMP16 | MMP3 | MPL | MRE11A |
| MRPL19 | MS4A13 | MSH2 | MSH3 | MSH6 | MTF1 |
| MTF2 | MTHFR | MTOR | MTR | MTRR | MUTYH |
| MYADM | MYB | MYC | MYCL | MYCN | MYD88 |
| MYO10 | MYOD1 | MYOM1 | MZT2A | NAB1 | NAB2 |
| NAMPT | NAPG | NAV1 | NBAS | NBEAL1 | NBN |
| NCOA6 | NCOR1 | NEDD4L | NEO1 | NF1 | NF2 |
| NFE2L2 | NFKBIA | NFXL1 | NKAP | NKX2-1 | NLRP7 |
| NOTCH1 | NOTCH2 | NOTCH3 | NOTCH4 | NPM1 | NR1I3 |
| NR4A3 | NRAS | NRG4 | NSD1 | NT5C2 | NTHL1 |
| NTRK1 | NTRK2 | NTRK3 | NUDT13 | NUP85 | NUP93 |
| OSBP | OTOGL | OTOS | P2RY8 | PAK1 | PAK7 |
| PALB2 | PAPOLG | PAQR8 | PARD6B | PRKN | PARP1 |
| PARP2 | PARP3 | PARP8 | PAX5 | PBRM1 | PDCD1 |
| PDCD1LG2 | PDE4D | PDGFB | PDGFRA | PDGFRB | PDPK1 |
| PDS5A | PFKP | PGBD1 | PGR | PGRMC2 | PHF20 |
| PIGF | PIK3C2G | PIK3C3 | PIK3CA | PIK3CB | PIK3CD |
| PIK3CG | PIK3R1 | PIK3R2 | PIK3R3 | PIM1 | PKHD1 |
| PLCG2 | PLEKHA1 | PLEKHH2 | PLXNC1 | PMS1 | PMS2 |
| PNO1 | POLA1 | POLD1 | POLE | POSTN | PPARG |
| PPP1R21 | PPP2R1A | PRDM1 | PRELID3B | PREX2 | PRKAR1A |
| PRKCI | PRKDC | PRPF39 | PRPF4 | PTCH1 | PTEN |
| PTK2 | PTPN11 | PTPN4 | PTPRD | PTPRJ | PTPRS |
| PTPRT | PURA | RAB2B | RABGAP1L | RAC1 | RAD21 |
| RAD50 | RAD51 | RAD51B | RAD51C | RAD51D | RAD52 |
| RAD54L | RAF1 | RALGAPB | RAP2B | RARA | RASA1 |
| RB1 | RBM10 | RBM27 | RECQL4 | REL | RET |
| RFC1 | COP1 | RHOA | RHOT1 | RIC1 | RICTOR |
| RIPK2 | RIT1 | RNF112 | RNF19A | RNF43 | ROBO1 |
| ROS1 | RPF2 | RPRD1A | RPTOR | RRM1 | RRP1B |
| RUNX1 | RWDD1 | RYBP | RYR2 | SASH1 | SCOC |
| SDHA | SDHAF2 | SDHB | SDHC | SDHD | SEL1L3 |
| SEMA3C | SEMA3E | SERTAD4 | SETD2 | SF3B1 | SFXN4 |
| SH2D1A | SHQ1 | SHROOM3 | SIMC1 | SIPA1L2 | SKA3 |
| SLC13A1 | SLC22A2 | SLC25A13 | SLC30A5 | SLC31A1 | SLC34A2 |
| SLC35B1 | SLC7A8 | SLC9C2 | SLCO1B1 | SLCO1B3 | SLIT1 |
| SLX4 | SMAD2 | SMAD3 | SMAD4 | SMARCA4 | SMARCB1 |
| SMO | SNX6 | SOCS1 | SOD2 | SOX17 | SOX2 |
| SOX9 | SPEN | SPOP | SRC | SRSF3 | SS18 |
| STAB2 | STAG2 | STARD4 | STAT3 | STK11 | STMN1 |
| STRBP | STT3A | STYX | SUCLG1 | SUFU | SUGCT |
| SUZ12 | SYK | SYNE2 | TAF15 | TAOK3 | TARBP1 |
| TBC1D8B | TBCD | TBX3 | TECPR2 | TENM3 | TERT |
| TET1 | TET2 | TFDP1 | TGFBR1 | TGFBR2 | TMEM126B |
| TMEM127 | TMEM132D | TMEM67 | TMPRSS15 | TMPRSS2 | TMTC4 |
| TNFAIP3 | TNFRSF14 | TNFSF13B | TNIK | TNKS | TNRC18 |
| TOP1 | TOP2B | TP53 | TP63 | TPH1 | TPM1 |
| TRA2A | TRAF7 | TRIM24 | TRIM25 | TSC1 | TSC2 |
| TSHR | TSN | TTC1 | TTN | TUBD1 | TXNDC16 |
| TXNRD1 | U2AF1 | UBAP2L | UBE2E3 | UBE4A | UBN2 |
| UBXN7 | UGT1A1 | ULK2 | ULK4 | UPF2 | USP11 |
| USP34 | UTS2 | VEGFA | VHL | VSIG10 | WDR5 |
| NSD2 | NSD3 | WT1 | XIAP | XPC | XPO1 |
| XRCC1 | XRCC2 | YAP1 | YLPM1 | YWHAE | ZBBX |
| ZBTB40 | ZDHHC17 | ZDHHC20 | ZMYM2 | ZMYM4 | ZNF195 |
| ZNF2 | ZNF280D | ZNF283 | ZNF367 | ZNF711 | ZNF805 |
| ZNF91 | ZZZ3 | CHRM3 | BCL2L11 | PAX3 | TFRC |
| FGF10 | NRG1 | FLI1 | FGF6 | TTC6 | CHD2 |
| RPS6KB1 | DNAJB1 | BCR | UMPS | SRY | USP9Y |
| UTY |  |  |  |  |  |
